# Meme-Based Packaging as Digital Cultural Translation: How Online Cultural Symbols Shape Purchase and Sharing Intentions

**DOI:** 10.3390/bs16060972

**Published:** 2026-06-11

**Authors:** Yuchen Song, Kiesu Kim

**Affiliations:** College of Design, Silla University, Busan 46958, Republic of Korea; syc9336@gmail.com

**Keywords:** meme-based packaging, packaging design, cultural resonance, perceived value, purchase intention, sharing intention, Stimulus–Organism–Response, PLS-SEM

## Abstract

Internet memes increasingly move from social media into physical product packaging, yet little is known about how consumers respond when online cultural symbols become package design cues. Drawing on the Stimulus–Organism–Response framework, this study examines how meme-based packaging shapes purchase intention and sharing intention through perceived value, brand warmth, and cultural resonance. A between-subjects survey experiment was conducted with 305 Chinese adult consumers, who evaluated either a meme-based packaging stimulus or a no-explicit-meme conventional packaging control stimulus. Partial least squares structural equation modeling showed that purchase intention and sharing intention followed different dominant mechanisms. Perceived value was the strongest predictor of purchase intention, whereas cultural resonance was the strongest predictor of sharing intention. Visual attractiveness most strongly enhanced perceived value, while playfulness and expression–product fit contributed more clearly to brand warmth and cultural resonance. Mediation results further showed that brand warmth and cultural resonance consistently transmitted the effects of meme-packaging cues, whereas the value route was more selective. These findings show how online cultural symbols can continue to shape consumer evaluation and social transmission after entering physical product interfaces.

## 1. Introduction

In China, meme-inflected products and packaging have increasingly become visible in fast-moving consumer goods, where online slang and character-based humor are used to attract attention and encourage social discussion. Internet memes have become a routine part of digital culture. They appear as images, captions, short videos, remixed slogans, expressive templates, and ironic references that circulate across social media platforms and everyday communication. Meme studies conceptualize memes as cultural units that are copied, modified, and recontextualized by users (Shifman, 2014; Dawkins, 1976; Jenkins et al., 2013). Contemporary memes also function as social signals, allowing users to express humor, cultural knowledge, group membership, and shared attitudes in compressed form. As brands increasingly participate in platform culture, memes have entered marketing communication, e-commerce promotion, influencer content, and consumer engagement strategies (Tsai & Hsiao, 2025; Kim & Baek, 2025).

In China, the relevance of meme-based packaging is closely connected with the everyday diffusion of short-video platforms, social networking services, and mobile media practices. The 55th Statistical Report on China’s Internet Development reported that short-video users in China reached 1.04 billion by December 2024, accounting for 93.8% of all internet users (China Internet Network Information Center, 2025). This indicates that platform-based visual language, humorous expression, and meme-like cultural cues have become part of ordinary digital media consumption rather than remaining within a narrow online subculture.

Most studies of meme marketing still focus on digital channels. This emphasis is understandable because memes originate and circulate most visibly online. However, a growing number of brands now translate meme language into physical product packaging. Snack, beverage, cosmetic, and lifestyle brands increasingly use online slang, playful captions, exaggerated character expressions, and culturally recognizable catchphrases on product packages. In these cases, the meme is not only a message posted by a brand. It becomes part of the package, and the package becomes a material carrier of digital cultural expression. This shift raises a theoretical problem. Packaging is not equivalent to a social media post. A meme on a platform can rely on comments, reposts, algorithmic exposure, sound, timing, and user remixing. Product packaging has a different communicative ecology. It must attract attention, communicate product information, signal quality, express brand personality, and support purchase decisions within a limited visual field (Orth & Malkewitz, 2008; Silayoi & Speece, 2007; Krishna et al., 2017). If a meme is simply attached to a package without regard to legibility, product fit, or visual organization, it may be perceived as superficial trend-chasing. If the meme expression is well translated, the package may communicate not only product value but also cultural belonging and shareability. This makes meme-based packaging a design translation problem rather than a simple extension of online meme marketing.

When internet memes move from social media feeds to product packaging, their meaning is no longer shaped by platform interaction alone. The package must make an online cultural sign recognizable at a glance, translate it into a coherent visual expression, and connect it to the product category without obscuring basic product information. We therefore approach meme-based packaging as a form of digital cultural translation: online cultural signs are reformulated as packaging cues that can be evaluated, interpreted, and potentially shared in an offline consumption context.

The present study asks how such packaging affects consumer purchase intention and sharing intention. These two outcomes are deliberately separated because purchase intention and sharing intention reflect different consumer responses. Purchase intention is a conversion-oriented outcome, whereas sharing intention is a transmission-oriented outcome. Research on word-of-mouth, online sharing, and valuable virality indicates that people share content because it is emotionally arousing, socially valuable, identity-relevant, or conversationally useful (Berger & Milkman, 2012; Berger, 2014; Akpinar & Berger, 2017). Meme-based packaging may therefore have a distinctive effect on sharing because it can turn a physical product package into socially meaningful content.

To explain this process, this study builds a Stimulus–Organism–Response (S-O-R) model. The stimulus layer consists of five perceived meme-packaging design cues: playfulness, information clarity, visual attractiveness, perceived novelty, and expression–product fit. The organism layer consists of perceived value, brand warmth, and cultural resonance. The response layer consists of purchase intention and sharing intention. This design enables the study to examine whether meme-based packaging works through a functional value route, an affinity route, and an expression–culture route.

This research makes three contributions. First, it extends meme-marketing research beyond online branded content by examining memes as packaging design resources. Second, it enriches packaging design research by introducing cultural resonance as a mechanism that explains social transmission, not only purchase evaluation. Third, it distinguishes the psychological foundations of purchase intention and sharing intention, offering a more precise account of how meme-based packaging may support both commercial evaluation and social circulation. This study has two connected aims: to conceptualize meme-based packaging as a design translation process and to explain how translated packaging cues influence purchase intention and sharing intention through different psychological mechanisms. By linking design translation with the S-O-R framework, the study examines how digital popular culture enters product evaluation and social communication through packaging.

## 2. Literature Review and Hypothesis Development

### 2.1. Meme-Based Packaging as Design Translation

Internet memes are now part of everyday online communication. Users create, adapt, and circulate memes through images, captions, short videos, and catchphrases (Shifman, 2014; Dawkins, 1976; Jenkins et al., 2013). Memes are not fixed messages; their meanings often emerge through repeated use, remixing, and shared recognition within platform communities. Brands have also increasingly adopted meme-like language, humor, and visual formats to attract attention, show cultural responsiveness, and encourage consumer interaction (Tsai & Hsiao, 2025; Kim & Baek, 2025). Recent studies suggest that such content can shape brand attitudes, consumer engagement, and purchase-related responses when it is humorous, timely, and compatible with platform culture (Appel et al., 2020; Rathi & Jain, 2024).

When memes move from social media into product packaging, the object of analysis changes. Packaging cannot rely on comments, reposts, hashtags, or platform context to complete meaning. Consumers instead encounter a fixed product interface and must judge product information, visual expression, and product fit within limited package space. Meme-based packaging is therefore better understood as a design translation process than as meme marketing placed on a package surface.

In this study, digital cultural translation describes how meme-related symbols from online culture are selected, integrated into package graphics, and aligned with the product category, brand tone, and consumption context. These operations matter because the package must make the meme reference recognizable without weakening visual hierarchy or product information. Meme-based packaging is therefore not playful surface decoration, but a consumer-facing redesign of online cultural language.

### 2.2. Stimulus–Organism–Response Framework in Packaging and Consumer Behavior

The S-O-R framework explains how external stimuli influence internal organism states, which then shape behavioral responses (Mehrabian & Russell, 1974). It has been widely used in digital interface and online retail settings (Z. Chen & Lee, 2025; Tian et al., 2022), food and organic-consumption contexts (Sultan et al., 2021; P.-J. Chen & Antonelli, 2020; Roh et al., 2022), and broader marketing and social commerce research (Eroglu et al., 2001; Mariani et al., 2022). In this study, the external stimuli are perceived packaging cues rather than objective design elements. This distinction is important because consumers respond to how the package is experienced, not only to how designers intended it.

The organism layer includes perceived value, brand warmth, and cultural resonance because these constructs capture different but complementary psychological responses to meme-based packaging. Perceived value represents the consumer’s evaluative judgment of whether the product and its package appear useful, worthwhile, and purchase-relevant (Zeithaml, 1988; Sweeney & Soutar, 2001). Brand warmth captures whether the playful and approachable expression makes the brand appear more human, friendly, and close to consumers (Aaker et al., 2010; Kervyn et al., 2012). Cultural resonance captures whether consumers recognize and connect with the digital cultural context embedded in the package. Together, these three constructs allow the model to distinguish value evaluation, relational brand perception, and cultural identification rather than treating consumer response as a single general attitude.

The response layer includes purchase intention and sharing intention. Prior consumer research often treats purchase intention as the primary outcome of packaging evaluation (Liu et al., 2025; Shukla et al., 2022). Design and package communication studies similarly connect package cues to product evaluation (Silayoi & Speece, 2007; Ampuero & Vila, 2006). However, in meme-based packaging, sharing intention is equally important because the package may operate as a social object. A consumer may share a package because it is emotionally engaging or socially valuable (Berger & Milkman, 2012; Berger, 2014), because it supports identity signaling (Chu & Kim, 2011; Buchanan, 2020), or because it provides conversational value in online interaction (Laato et al., 2020; Lindstrom et al., 2021). Thus, the present model differentiates between a decision response and a transmission response.

The S-O-R framework explains how external stimuli influence an organism’s internal state, thereby shaping behavioral responses (Mehrabian & Russell, 1974). Leveraging the integrative advantages of the S-O-R framework, this study constructs a “dual stimulus-dual-path mediation-social response” model: packaging design and meme marketing (S) trigger symbolic consumption cognition and brand emotion (O), which in turn drive social communication behavior (R), revealing the psychological mechanisms and pathways by which packaging design empowers brand social communication in the context of meme marketing. The theoretical foundations and cue-route mapping of the proposed model are summarized in Table 1.

This separation is also consistent with the broader logic of behavioral intention. Intention is not a neutral afterthought; it summarizes motivational readiness to perform a behavior and is therefore shaped by the beliefs and meanings that consumers attach to an object (Ajzen, 1991). In a packaging context, the same stimulus can support more than one behavioral intention. A package may strengthen purchase intention by increasing perceived product benefits, while strengthening sharing intention by increasing expressive or conversational value. Creative consumption research similarly suggests that experiential response and purchase response do not always move for identical reasons (Aucouturier et al., 2015). For meme-based packaging, this distinction is especially important because the design may be evaluated simultaneously as a product cue, a brand cue, and a piece of culturally recognizable content.

### 2.3. Functional Value Path: Information Clarity, Visual Attractiveness, Expression–Product Fit, and Perceived Value

Perceived value refers to consumers’ overall judgment of whether a product is worth choosing, considering what they receive and what they give up (Zeithaml, 1988; Sweeney & Soutar, 2001). In packaging contexts, this judgment is shaped not only by the product itself but also by how the package communicates information, presents visual quality, and connects its expression with the product. This issue becomes especially important for meme-based packaging. A package may be playful and attention-grabbing, but consumers still need to understand what the product is, why the design is relevant, and whether the product appears worth buying.

Information clarity is therefore a basic condition for value perception. Meme expressions are often compressed, ironic, and context-dependent. If they obscure product information or make the package difficult to understand, consumers may enjoy the humor but hesitate to infer product value. Clear information can reduce cognitive effort and perceived uncertainty, especially when packaging uses unconventional or playful expressions (Silayoi & Speece, 2007; Krishna et al., 2017; Magnier & Schoormans, 2015). When meme expression and product information remain coordinated, consumers can appreciate the playful design without losing the functional clarity needed for evaluation.

Visual attractiveness is another important source of perceived value. Packaging is usually evaluated quickly, and visual quality can shape first impressions of product desirability and design effort (Liu et al., 2025; Bloch, 1995; Ampuero & Vila, 2006). For meme-based packaging, visual attractiveness does not only mean beauty or color harmony. It also concerns whether meme elements, typography, imagery, and layout are organized into a coherent visual system. A visually attractive package can make meme expression appear more intentional and better designed, thereby strengthening consumers’ perception of product value.

Expression–product fit captures whether the meme expression seems appropriate for the product category, consumption situation, and brand voice. A meme that fits the product is more likely to be interpreted as purposeful rather than randomly attached for attention. Schema congruity research suggests that consumers evaluate new cues more favorably when they can be integrated into existing expectations about the product (Meyers-Levy & Tybout, 1989). In meme-based packaging, such fit can help consumers understand why the meme belongs with the product, which may further support perceived value.

Based on the above reasoning, the following hypotheses are proposed:

**H1a.**  *In the context of meme-based packaging, information clarity positively affects perceived value.*

**H1b.**  *In the context of meme-based packaging, visual attractiveness positively affects perceived value.*

**H1c.**  *In the context of meme-based packaging, expression–product fit positively affects perceived value.*

Perceived value is also expected to influence purchase intention. Consumers may find a meme package amusing or culturally interesting, but purchase decisions still depend on whether the product appears useful, desirable, or worth trying. Prior consumer research has consistently treated perceived value as a key antecedent of purchase-related responses (Zeithaml, 1988; Sweeney & Soutar, 2001). In the present context, meme-based packaging is more likely to support purchase intention when consumers perceive that the product and its packaging offer sufficient value.

Accordingly, the following hypothesis is proposed:

**H2.**  *Perceived value positively affects purchase intention toward the evaluated packaged product.*

The functional value path further suggests a mediation process. Information clarity, visual attractiveness, and expression–product fit may not influence purchase intention only by attracting attention. They may first strengthen consumers’ perceived value and then increase their willingness to buy or try the product. This mediation logic is especially relevant for meme-based packaging because playful expression alone is unlikely to support purchase unless it also contributes to a sense of product value.

Thus, the following hypothesis is proposed:

**H3.**  *Perceived value mediates the effects of information clarity, visual attractiveness, and expression–product fit on purchase intention in the context of meme-based packaging.*

### 2.4. Affinity Path: Playfulness, Visual Attractiveness, Expression–Product Fit, and Brand Warmth

Meme-based packaging may also influence consumer response by changing how the brand feels. Compared with conventional packaging, which often relies on formal or promotional language, meme-based packaging tends to use a more casual, humorous, and socially familiar style. When this shift is successful, the brand may appear less distant and more human. The concept of brand warmth is relevant here because it captures perceptions of friendliness, sincerity, and approachability (Aaker et al., 2010; Kervyn et al., 2012). Prior research on brand relationships further suggests that such impressions can shape consumer closeness and trust (Fiske et al., 2007; Fournier, 1998).

Related work on brand authenticity and brand communication also suggests that sincerity and credibility can shape consumer evaluations of fast-moving consumer goods (Morhart et al., 2015; Dwivedi & McDonald, 2018).

Playfulness is likely to be an important antecedent of brand warmth. Research on hedonic consumption shows that fun, fantasy, and emotional experience play a central role in consumer response (Holbrook & Hirschman, 1982). Humor-related research also suggests that amusement can influence social interaction and relational evaluation (Warren et al., 2018). In the context of meme-based packaging, playful expression may reduce psychological distance and make the brand feel less rigid and more approachable.

Visual attractiveness may also strengthen brand warmth. A well-designed package does more than create aesthetic pleasure; it can also signal care, effort, and attention to consumer experience (Bloch, 1995; Velasco & Spence, 2019). For meme-based packaging, visual attractiveness is especially important because humorous or culturally coded content can easily appear messy or excessive if it is not visually organized. When meme elements are integrated into a clear and appealing design, consumers may be more likely to perceive the brand as thoughtful and considerate.

Expression–product fit is another likely source of brand warmth. Consumers may respond more positively when the meme expression feels naturally connected to the product, the usage context, and the brand’s tone of voice. If the fit is weak, the package may seem forced or opportunistic. If the fit is strong, the same expression may signal that the brand understands both its product and the consumer’s cultural language. This kind of appropriateness can make the brand feel more relatable and closer to the consumer.

Based on the above reasoning, the following hypotheses are proposed:

**H4a.**  *In the context of meme-based packaging, playfulness positively affects brand warmth.*

**H4b.**  *In the context of meme-based packaging, visual attractiveness positively affects brand warmth.*

**H4c.**  *In the context of meme-based packaging, expression–product fit positively affects brand warmth.*

Brand warmth is further expected to influence both purchase intention and sharing intention. Warm brands are generally more likely to be liked and trusted (Aaker et al., 2010; Kervyn et al., 2012). They may also be easier for consumers to incorporate into self-expression when the brand feels familiar, credible, and emotionally close (Fiske et al., 2007; Fournier, 1998). In the case of meme-based packaging, if the packaging makes the brand appear friendly and culturally fluent, consumers may become more willing not only to try the product but also to show the package to others.

Accordingly, the following hypotheses are proposed:

**H5a.**  *Brand warmth positively affects purchase intention toward the evaluated packaged product.*

**H5b.**  *Brand warmth positively affects sharing intention toward the evaluated packaged product.*

This path also implies a mediation process. Playfulness, visual attractiveness, and expression–product fit may shape consumer responses not only because the package looks appealing or feels entertaining, but also because these cues make the brand seem warmer and more approachable. In this sense, brand warmth serves as an important relational mechanism linking meme-based packaging with both purchase and sharing responses.

Thus, the following hypothesis is proposed:

**H6.**  *Brand warmth mediates the effects of playfulness, visual attractiveness, and expression–product fit on purchase intention and sharing intention in the context of meme-based packaging.*

### 2.5. Expression–Culture Path: Playfulness, Perceived Novelty, Expression–Product Fit, and Cultural Resonance

The expression–culture path explains what makes meme-based packaging different from conventional creative packaging. A package can be visually novel without being culturally resonant. Meme-based packaging, however, depends on whether consumers recognize the digital cultural context behind the expression. Cultural resonance occurs when consumers understand the meme reference, connect it with familiar online language or platform culture, and perceive it as socially meaningful.

This mechanism is grounded in the idea that products and brands carry symbolic meanings beyond functional utility (Belk, 1988; McCracken, 1986; Holt, 1995). Consumers may use culturally coded products not only to satisfy practical needs, but also to express taste, identity, and shared belonging. Studies on cultural products, authenticity, and symbolic consumption show that consumers respond more positively when product meanings connect with their cultural interests, self-expression, or sense of identification (Li et al., 2023; Kolar & Zabkar, 2010; He et al., 2023). Meme-based packaging reflects a newer form of this symbolic process. Its cultural references do not come from traditional heritage alone, but from digital popular culture: jokes, catchphrases, visual templates, and platform-specific ways of speaking.

Playfulness is likely to increase cultural resonance because humor and lightness are central to many internet memes. When consumers recognize the playful expression, they may not only find the package amusing, but also feel that it belongs to a shared online context. In this sense, playfulness is not only an emotional cue; it can also serve as an entry point for cultural recognition.

Perceived novelty may also strengthen cultural resonance. Internet memes are sensitive to timing, freshness, and cultural momentum. A package that feels different from ordinary packaging may signal that the brand is responding to current digital culture rather than repeating familiar promotional language. When novelty is perceived as meaningful and culturally timely, consumers are more likely to see the package as part of a living social conversation.

Expression–product fit further supports cultural resonance. Not every meme can be naturally translated into packaging. If the meme expression feels disconnected from the product, consumers may see it as forced or opportunistic. If it fits the product category, consumption situation, and brand voice, the same expression becomes easier to accept as a meaningful design choice. Fit therefore helps transform a meme from a borrowed online joke into a culturally coherent packaging cue.

Based on the above reasoning, the following hypotheses are proposed:

**H7a.**  *In the context of meme-based packaging, playfulness positively affects cultural resonance.*

**H7b.**  *In the context of meme-based packaging, perceived novelty positively affects cultural resonance.*

**H7c.**  *In the context of meme-based packaging, expression–product fit positively affects cultural resonance.*

Cultural resonance may then influence both purchase intention and sharing intention. When consumers feel that a package expresses meanings connected with their own cultural experience or social world, they may become more willing to try the product. However, the role of cultural resonance should be especially important for sharing intention. Prior research on word-of-mouth, online sharing, and virality suggests that people share content when it is emotionally engaging, socially valuable, identity-relevant, or useful for conversation (Berger & Milkman, 2012; Berger, 2014; Chu & Kim, 2011). A culturally resonant meme package gives consumers something to show to others: not merely a product, but a recognizable sign that can communicate humor, belonging, or shared understanding.

Accordingly, the following hypotheses are proposed:

**H8a.**  *Cultural resonance positively affects purchase intention toward the evaluated packaged product.*

**H8b.**  *Cultural resonance positively affects sharing intention toward the evaluated packaged product.*

This path also implies a mediation process. Playfulness, perceived novelty, and expression–product fit may influence consumer responses because they help the package generate cultural resonance. This mediation is especially relevant for sharing intention, as consumers are more likely to share a package when it feels culturally recognizable and socially meaningful, rather than merely attractive or unusual.

Thus, the following hypothesis is proposed:

**H9.**  *Cultural resonance mediates the effects of playfulness, perceived novelty, and expression–product fit on purchase intention and sharing intention in the context of meme-based packaging.*

### 2.6. Research Model

Drawing on the preceding hypotheses, this study proposes a three-route S-O-R model of meme-based packaging. The model includes five stimulus variables, namely playfulness, information clarity, visual attractiveness, perceived novelty, and expression–product fit; three organism variables, namely perceived value, brand warmth, and cultural resonance; and two response variables, namely purchase intention and sharing intention.

**Figure 1:**
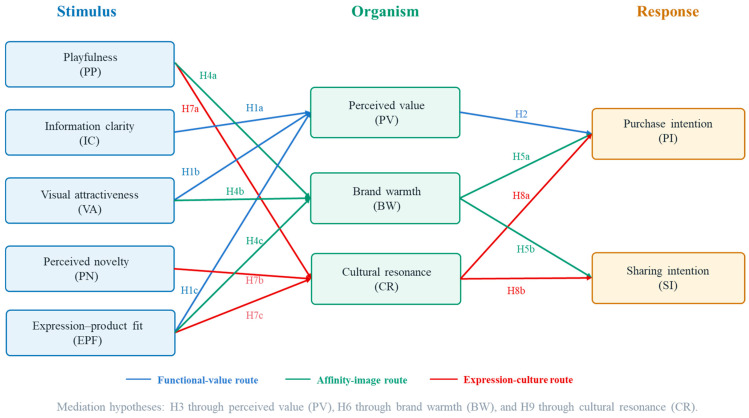
Proposed design translation S-O-R model of meme-based packaging.

The functional value route explains how information clarity, visual attractiveness, and expression–product fit shape purchase intention through perceived value. The affinity–image route explains how playfulness, visual attractiveness, and expression–product fit influence purchase and sharing intentions through brand warmth. The expression–culture route explains how playfulness, perceived novelty, and expression–product fit influence purchase and sharing intentions through cultural resonance. These route names are shown in Figure 1 to make the model structure clearer.

The model therefore treats meme-based packaging not only as a product information carrier, but also as a brand cue and a culturally recognizable design expression. The core hypotheses focus on these psychological mechanisms, whereas additional checks are reported in the Methods and Supplementary Materials as robustness analyses rather than as central theoretical hypotheses. Figure 1 presents the proposed research model.

## 3. Materials and Methods

### 3.1. Research Design and Stimulus Materials

This study used a between-subjects survey experiment combined with a cross-sectional questionnaire to examine how meme-based packaging cues influence consumers’ psychological responses and behavioral intentions. Two packaging stimulus conditions were developed: a meme-based packaging condition and a no-explicit-meme conventional packaging control condition. The meme-based condition included a meme-like cat character, playful visual expression, and humorous copy associated with online popular culture. The conventional control condition removed the explicit meme character, meme-style copy, and digital cultural visual cues and replaced them with more conventional product images and ordinary product-related expressions.

The focal product context was the Joyoung “Hakimi North–South Mung Bean” soy milk drink, a 150 g pouch-format plant-based beverage. This product context was selected because meme-based packaging had become visible in online consumer discussion and because the pouch format provided sufficient visual space to manipulate meme expression while keeping product category, package format, brand placement, basic layout, and core product information comparable across conditions.

The conventional packaging control condition retained the main product identification elements, including the brand position, product name, 150 g net-content statement, ingredient-related imagery, and basic beverage-consumption cues. It removed the explicit meme character, meme-style copy, and digital cultural visual cues and presented a more product-centered package design.

The meme-based packaging condition used the same product and package format but made the “Hakimi” meme reference explicit within the package interface. It incorporated cat character imagery, internet-style captions, exaggerated humorous scenes, and stronger visual personality cues while retaining the product name, brand position, package format, and basic product information.

Because a real marketplace brand context was used, brand familiarity was measured and included as a control variable in the supplementary robustness check. The final group sizes were similar, with 151 respondents in the meme-based packaging condition and 154 respondents in the conventional packaging control condition.

The two stimuli were designed to compare a package with explicit meme-based visual and textual cues against a no-explicit-meme conventional control while keeping the core product context comparable. The conventional control removed the explicit meme character, meme-style copy, and digital cultural visual cues, while retaining the same focal product, package structure, and basic product information.

Both stimuli were presented before the measurement items in the online survey. The product category, package format, brand information, and basic product information were kept comparable so that the experimental manipulation primarily concerned the intensity and salience of meme expression. A manipulation check was included to assess whether respondents perceived the two stimuli as differing in meme intensity.

**Figure 2:**
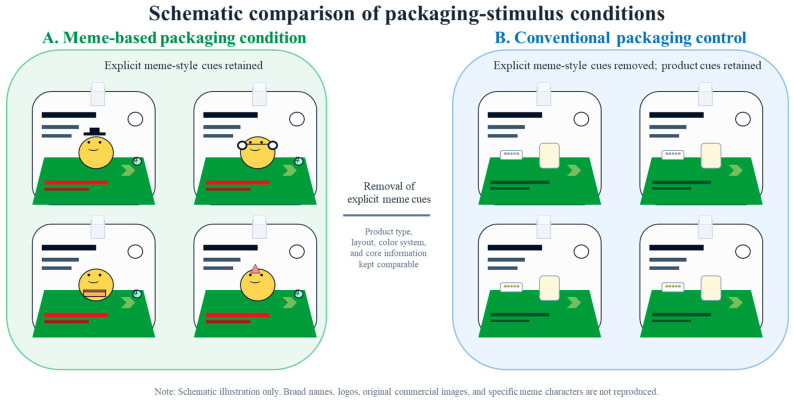
Schematic illustration of the stimulus materials.

For copyright reasons, the original commercial package images are not reproduced in full detail in the manuscript or Supplementary Materials. Instead, Figure 2 provides an anonymized schematic illustration of the two stimulus conditions, focusing on the presence or absence of meme-like characters, playful copy, and digital cultural visual cues.

### 3.2. Participants and Data Collection

The target population consisted of Chinese adult consumers aged 18 years and above. Participants were recruited through Wenjuanxing (Questionnaire Star), a widely used online survey platform in China, using a non-probability online sampling approach in April 2026. They completed one of the two questionnaire versions corresponding to the meme-based packaging condition or the conventional packaging control condition. This study did not define meme-based packaging as a phenomenon limited to a single generational cohort. Instead, it treated meme-based packaging as a broader design and consumer-response phenomenon in which digital popular culture symbols enter everyday product packaging.

Before answering the main questionnaire, participants first read an informed-consent statement. The statement explained the academic purpose of the study, the anonymous nature of the survey, voluntary participation, minimal risk, and the right to withdraw at any time. No personally identifiable information, such as names, phone numbers, email addresses, or ID numbers, was collected. Participants who agreed to continue then completed the screening questions, viewed the assigned packaging stimulus, and answered the main questionnaire items.

A total of 358 responses were initially collected. Data screening was conducted according to predefined quality-control rules. Responses were excluded if they failed the attention check or contained invalid values outside the 1–7 range for the core scale items. After screening, 305 valid responses were retained, yielding a valid response rate of 85.20%. Age was not used as an exclusion criterion except for the requirement that participants be at least 18 years old. The data screening procedure is summarized in Table 2.

The final sample covered multiple adult age groups rather than a single youth segment. The largest age group was 31–40 years (37.0%), followed by 25–30 years (29.2%) and 41–50 years (28.2%). Most respondents had junior college or bachelor’s degree education. Purchase frequency for fast-moving consumer goods was relatively high, with 42.3% reporting purchases five or more times per week. Gender was included as an optional demographic item; respondents who selected “not disclosed” were retained because this response reflected voluntary non-disclosure rather than a data-quality problem. Gender was not used as a focal construct in the main model. Table 3 presents the sample profile and group distribution.

### 3.3. Face Validity, Content Validity, and Pilot Test

The measurement items were adapted from prior studies and contextualized to the meme-based packaging setting. To support content validity, the initial item pool was reviewed against the definitions of each construct, including playfulness, information clarity, visual attractiveness, perceived novelty, expression–product fit, perceived value, brand warmth, cultural resonance, purchase intention, and sharing intention. Face validity was assessed by checking whether the items were understandable to ordinary consumers and whether the stimulus presentation matched the intended meme-based and conventional control conditions.

Before the formal survey, a pilot test was conducted with 100 adult participants, including 50 participants for the meme-based packaging questionnaire and 50 participants for the conventional packaging control questionnaire. The pilot test was used to assess item clarity, stimulus comprehension, approximate completion time, and the distinguishability of the two packaging conditions. Based on the pilot feedback, minor wording adjustments were made to improve item clarity and contextual fit, but no construct or measurement dimension was substantially changed. The pilot responses were not included in the final formal sample.

### 3.4. Measures

All focal constructs were measured using seven-point Likert scales, ranging from 1 = strongly disagree to 7 = strongly agree. The measurement items were adapted from prior studies and rewritten to fit the meme-based packaging context. The pilot test described above was also used to check item clarity, completion time, and respondents’ understanding of the stimulus materials.

Packaging playfulness measured the extent to which the package was perceived as humorous, lighthearted, and entertaining. Information clarity measured whether respondents could clearly understand the product information and design expression presented on the package. Visual attractiveness measured the aesthetic appeal of the package in terms of graphics, color, typography, and layout. Perceived novelty measured whether the package was perceived as fresh, distinctive, and different from ordinary packaging. Expression–product fit measured whether the meme-like expression was perceived as appropriate for the product, brand tone, and consumption occasion.

Perceived value measured respondents’ overall evaluation of whether the product and its packaging were worthwhile. Brand warmth measured whether the package made the brand seem friendly, approachable, sincere, and human. Cultural resonance measured whether respondents recognized and connected with the digital cultural context expressed in the package. Purchase intention measured respondents’ willingness to buy or try the product. Sharing intention measured their willingness to photograph, discuss, forward, or share the package with others.

Table 4 summarizes the focal constructs, abbreviations, number of items, and main source domains. Full item wording is provided in Supplementary Table S1.

The constructs were modeled as reflective measures because the items were intended to capture different manifestations of the same underlying perception or intention. Reliability and validity were assessed in the measurement-model evaluation, following established PLS-SEM reporting recommendations.

### 3.5. Data Analysis

Data were analyzed using IBM SPSS Statistics 26.0 (IBM Corp., Armonk, NY, USA) and SmartPLS 4 (SmartPLS GmbH, Oststeinbek, Germany). SPSS was used for data screening, descriptive statistics, manipulation-check analysis, and supplementary robustness checks based on construct scores. SmartPLS 4 was used to assess the measurement model, estimate the structural model, test mediation effects, and conduct importance–performance map analysis.

PLS-SEM was used because the proposed model includes multiple packaging cues, three psychological mechanisms, and two behavioral outcomes. This approach is suitable for examining prediction-oriented models and for linking structural results with design implications through IPMA (Hair et al., 2019; Hair et al., 2021; Sarstedt et al., 2022). The analysis followed established PLS-SEM reporting procedures.

Several diagnostic procedures were conducted before interpreting the structural model. Reliability and convergent validity were assessed using indicator loadings, Cronbach’s alpha, composite reliability, and average variance extracted. Discriminant validity was assessed using HTMT (Henseler et al., 2015). Common method bias was examined through Harman’s single-factor test, full collinearity VIF, and a CFA comparison between a one-factor model and the hypothesized ten-factor measurement model. Structural paths and mediation effects were tested using bootstrapping with 10,000 subsamples and two-tailed tests, with mediation interpreted through bootstrapped indirect effects (Nitzl et al., 2016). IPMA was used to identify constructs with high importance but relatively lower performance for purchase intention and sharing intention.

The robustness model included age, gender, education, purchase frequency, price sensitivity, brand familiarity, and prior purchase experience as background variables. Because gender was collected as an optional demographic item, respondents who chose not to disclose gender were retained and coded as a separate category for the robustness check.

A CFA model comparison was also conducted as an additional diagnostic analysis and is reported in the common method bias assessment.

### 3.6. Common Method Bias Considerations

Because the study used a single self-report questionnaire at one point in time, several procedures were used to reduce and assess potential common method bias. Procedurally, the survey was anonymous; participants were informed that there were no right or wrong answers, and all responses were used only for academic research. The questionnaire also separated stimulus exposure, packaging design perceptions, psychological evaluations, behavioral intentions, and demographic items to reduce respondents’ tendency to answer related constructs in a single response pattern.

Statistical diagnostics were conducted after data collection. Harman’s single-factor test was used as an initial diagnostic. In addition, full collinearity VIF values were calculated based on construct scores, and a CFA model comparison was conducted between a one-factor model and the hypothesized ten-factor measurement model. These diagnostics were used together to assess whether a single common method factor was likely to account for the covariance among the focal measurement items. The detailed results are reported in the Results section and Supplementary Table S4.

## 4. Results

### 4.1. Effectiveness of the Meme-Intensity Manipulation

A manipulation check was conducted to verify whether the two stimulus conditions differed in perceived meme intensity. Respondents in the meme-based packaging condition reported a higher meme perception score (M = 5.31, SD = 1.14) than those in the conventional packaging control condition (M = 4.14, SD = 1.22). The difference was statistically significant, t(303) = 8.64, *p* < 0.001, with a large effect size, Cohen’s d = 0.99. These results indicate that the stimulus manipulation successfully produced a clear relative difference in perceived meme expression. However, because the conventional control condition still scored slightly above the scale midpoint, it should be interpreted as a no-explicit-meme conventional control rather than as evidence of an absolute absence of meme perception. The manipulation-check results are reported in Table 5.

### 4.2. Measurement Model Assessment

The reflective measurement model was assessed in terms of indicator reliability, internal consistency, convergent validity, and discriminant validity. As shown in Table 6, all outer loadings exceeded 0.90. Cronbach’s alpha ranged from 0.901 to 0.925, composite reliability (rho_c) ranged from 0.938 to 0.952, and AVE ranged from 0.835 to 0.870. These values exceeded the recommended thresholds, indicating satisfactory reliability and convergent validity.

Discriminant validity was assessed using the heterotrait–monotrait ratio (HTMT). All HTMT values were below 0.85. The highest HTMT value was observed between purchase intention and perceived value (0.826), which remained below the recommended threshold. The results therefore support discriminant validity among the constructs.

### 4.3. Common Method Bias Assessment

Because this study relied on a single self-report questionnaire, common method bias was assessed using multiple diagnostics (Podsakoff et al., 2003). Harman’s single-factor test was first conducted as an initial diagnostic. The first unrotated factor explained 60.25% of the total variance, suggesting a potential common method concern. Therefore, additional diagnostics were conducted rather than dismissing the issue on the basis of a single test.

A CFA model comparison was then performed. The one-factor model showed poor fit, χ^2^ = 2332.519, df = 405, χ^2^/df = 5.759, CFI = 0.786, TLI = 0.771, RMSEA = 0.125, and SRMR = 0.062. In contrast, the hypothesized ten-factor measurement model showed substantially better fit, χ^2^ = 401.124, df = 360, χ^2^/df = 1.114, CFI = 0.995, TLI = 0.994, RMSEA = 0.019, and SRMR = 0.022. These results indicate that a single common method factor did not adequately account for the covariance structure of the focal measurement items. Thus, although common method bias cannot be completely ruled out, it is unlikely to fully explain the observed structural relationships.

### 4.4. Structural Model Assessment

After the measurement model was confirmed, the structural model was assessed by examining predictor collinearity, explanatory power, path coefficients, effect sizes, and bootstrapped significance levels. Before testing the hypothesized paths, multicollinearity among the predictors was assessed using inner VIF values. All inner VIF values ranged from 1.599 to 2.709, below the conservative threshold of 3.3, indicating that multicollinearity was not a serious concern in the structural model.

The model showed substantial explanatory power for the endogenous constructs. The R^2^ values were 0.601 for perceived value, 0.613 for brand warmth, 0.634 for cultural resonance, 0.657 for purchase intention, and 0.563 for sharing intention. These values indicate that the model explained a meaningful proportion of variance in both the psychological mechanisms and behavioral outcomes.

**Figure 3:**
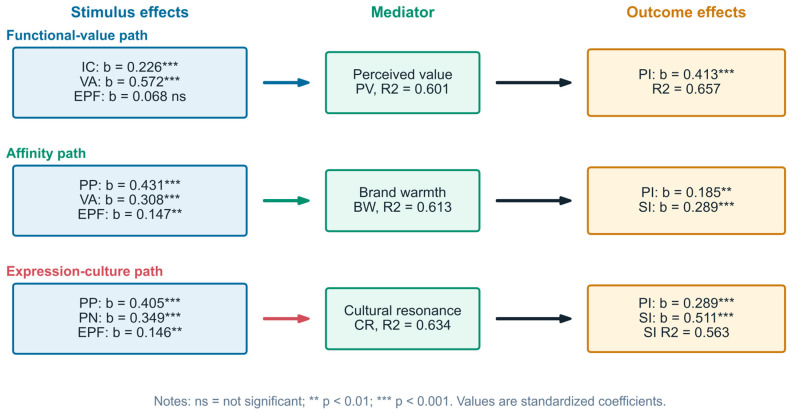
Structural model results. Values are standardized path coefficients.

The direct path results are reported in Table 7 and visualized in Figure 3. For the functional value path, information clarity had a significant positive effect on perceived value (β = 0.226, *p* < 0.001), and visual attractiveness showed a strong positive effect on perceived value (β = 0.572, *p* < 0.001). However, expression–product fit did not significantly affect perceived value (β = 0.068, *p* = 0.186). Therefore, H1a and H1b were supported, whereas H1c was not supported. By contrast, expression–product fit significantly predicted both brand warmth (β = 0.147, *p* = 0.001) and cultural resonance (β = 0.146, *p* = 0.001), suggesting that fit operated more clearly through relational and cultural mechanisms than through perceived value.

For the affinity–image path, playfulness had a significant positive effect on brand warmth (β = 0.431, *p* < 0.001), visual attractiveness had a significant positive effect on brand warmth (β = 0.308, *p* < 0.001), and expression–product fit also had a significant positive effect on brand warmth (β = 0.147, *p* = 0.001). Thus, H4a, H4b, and H4c were supported.

For the expression–culture path, playfulness had a significant positive effect on cultural resonance (β = 0.405, *p* < 0.001), perceived novelty had a significant positive effect on cultural resonance (β = 0.349, *p* < 0.001), and expression–product fit had a significant positive effect on cultural resonance (β = 0.146, *p* = 0.001). Thus, H7a, H7b, and H7c were supported.

Regarding the outcome paths, perceived value had a significant positive effect on purchase intention (β = 0.413, *p* < 0.001), supporting H2. Brand warmth significantly influenced both purchase intention (β = 0.185, *p* = 0.001) and sharing intention (β = 0.289, *p* < 0.001), supporting H5a and H5b. Cultural resonance also had significant positive effects on purchase intention (β = 0.289, *p* < 0.001) and sharing intention (β = 0.511, *p* < 0.001), supporting H8a and H8b.

Overall, the results reveal an asymmetric response pattern. Purchase intention was most strongly associated with perceived value, whereas sharing intention was most strongly associated with cultural resonance. Expression–product fit did not directly strengthen perceived value, but it significantly contributed to both brand warmth and cultural resonance. This pattern suggests that fit between meme expression and product context worked less as a functional value cue and more as a relational and cultural integration cue.

The pattern of results shows that purchase intention and sharing intention were associated with different dominant predictors. Perceived value was the strongest direct predictor of purchase intention, whereas cultural resonance was the strongest direct predictor of sharing intention. Effect sizes also showed that visual attractiveness had a large effect on perceived value (f^2^ = 0.474), cultural resonance had a medium-to-strong effect on sharing intention (f^2^ = 0.272), and playfulness had medium effects on brand warmth (f^2^ = 0.236) and cultural resonance (f^2^ = 0.201).

### 4.5. Mediation Analysis

Mediation analysis was conducted to examine the three proposed psychological pathways. The results are reported in Table 8. In the functional value path, information clarity had a significant indirect effect on purchase intention through perceived value (β = 0.093, *p* < 0.001), and visual attractiveness also had a significant indirect effect through perceived value (β = 0.237, *p* < 0.001). However, the indirect effect of expression–product fit on purchase intention through perceived value was not significant (β = 0.028, *p* = 0.195). Thus, H3 was partially supported.

In the affinity path, all indirect effects through brand warmth were significant. Playfulness indirectly affected purchase intention (β = 0.080, *p* = 0.002) and sharing intention (β = 0.124, *p* < 0.001) through brand warmth. Visual attractiveness indirectly affected purchase intention (β = 0.057, *p* = 0.012) and sharing intention (β = 0.089, *p* = 0.002) through brand warmth. Expression–product fit also had significant indirect effects on purchase intention (β = 0.027, *p* = 0.021) and sharing intention (β = 0.043, *p* = 0.011) through brand warmth. Therefore, H6 was supported.

In the expression–culture path, all indirect effects through cultural resonance were significant. Playfulness indirectly affected purchase intention (β = 0.117, *p* < 0.001) and sharing intention (β = 0.207, *p* < 0.001) through cultural resonance. Perceived novelty indirectly affected purchase intention (β = 0.101, *p* < 0.001) and sharing intention (β = 0.178, *p* < 0.001) through cultural resonance. Expression–product fit also had significant indirect effects on purchase intention (β = 0.042, *p* = 0.010) and sharing intention (β = 0.074, *p* = 0.002) through cultural resonance. Thus, H9 was supported.

### 4.6. Control Variable Robustness Check

To examine whether the main findings were robust to background characteristics, a supplementary model added age, gender, education, purchase frequency, price sensitivity, brand familiarity, and prior purchase experience as control variables.

After the seven background variables were added, the R^2^ value for purchase intention increased slightly from 0.657 to 0.666, and the R^2^ value for sharing intention increased from 0.563 to 0.572. The central effects of perceived value, brand warmth, and cultural resonance remained significant.

### 4.7. Importance–Performance Map Analysis

**Figure 4:**
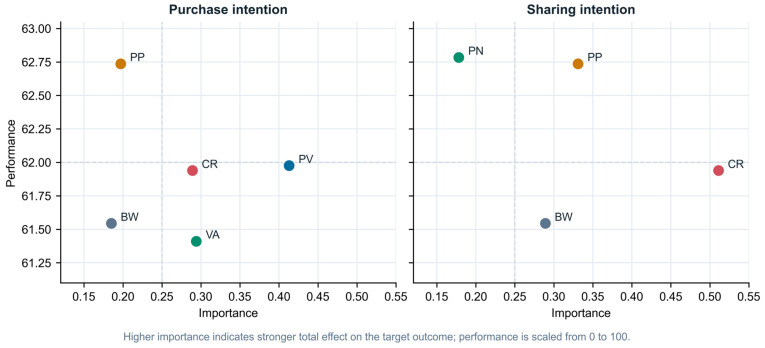
Importance–performance map analysis priorities for purchase intention and sharing intention.

Importance–performance map analysis (IPMA) was conducted to identify the relative design priorities associated with purchase intention and sharing intention. To keep the IPMA table focused on actionable priorities, Table 9 reports constructs with importance values of 0.15 or above for each target outcome. Lower-importance indirect effects for sharing intention, such as expression–product fit (approximately 0.117) and visual attractiveness (approximately 0.089), were therefore not listed as priority constructs. The results are summarized in Table 9 and visualized in Figure 4.

For purchase intention, perceived value showed the highest importance (0.413) with a performance score of 61.976, making it the highest-priority construct. Visual attractiveness also showed relatively high importance (0.294) with a performance score of 61.411. Cultural resonance had a similar level of importance (0.289) and a performance score of 61.939. Playfulness and brand warmth showed lower importance values, although both remained relevant to purchase intention.

For sharing intention, cultural resonance was the highest-priority construct, with an importance value of 0.511 and a performance score of 61.939. Playfulness was the second most important factor (importance = 0.331, performance = 62.737), followed by brand warmth (importance = 0.289, performance = 61.544). Perceived novelty showed lower importance than these constructs (importance = 0.178), although its performance score was relatively high (62.784).

Overall, the IPMA results indicate that purchase intention and sharing intention were associated with different priority structures. Perceived value was the most important construct for purchase intention, whereas cultural resonance was the most important construct for sharing intention. This distinction is important for design decision-making because it suggests that purchase-oriented optimization and sharing-oriented optimization should not follow the same design priorities.

## 5. Discussion

### 5.1. Key Findings and Interpretation

This study examined how meme-based packaging influences consumer purchase intention and sharing intention through three psychological mechanisms: perceived value, brand warmth, and cultural resonance. The findings suggest that meme-based packaging should not be understood merely as a playful packaging tactic. Rather, it works as a form of digital cultural translation in which online cultural symbols are transformed into packaging cues that consumers can evaluate, relate to, and potentially share.

The first key finding is that meme-based packaging does not operate through a simple “fun-to-buy” mechanism. Purchase intention and sharing intention were driven by different dominant mechanisms: perceived value was the strongest predictor of purchase intention, whereas cultural resonance was the strongest predictor of sharing intention. Buying a packaged beverage remains closely tied to value judgment, because consumers need to feel that the product is clear, appealing, and worth trying. Sharing, however, is closer to social expression, because consumers may share a package when it activates a recognizable cultural language that can be displayed, discussed, or circulated in social interaction. This distinction is consistent with the theoretical separation between value-oriented conversion and socially oriented transmission developed in the preceding sections.

The second key finding concerns visual attractiveness. Visual attractiveness had the strongest effect on perceived value. This result suggests that meme-based packaging cannot rely on meme expression alone. A package may contain a recognizable joke, phrase, or character, but if the design is visually weak, cluttered, or poorly organized, it may not help consumers form a positive value judgment. For meme-based packaging to support purchase-related evaluation, meme elements still need to be integrated into a coherent visual system. This finding is consistent with packaging and product design research showing that visual form and design quality influence consumer evaluation (Bloch, 1995; Shukla et al., 2022).

The third key finding concerns expression–product fit. The non-significant EPF → PV path deserves careful interpretation. This result does not mean that expression–product fit is unimportant in meme-based packaging. Rather, it suggests that fit may not operate as a direct value enhancement cue in this product context. For a low-involvement packaged beverage, consumers’ perceived value appears to depend more strongly on whether the package is visually attractive and whether the product information remains clear. However, expression–product fit significantly predicted both brand warmth and cultural resonance, indicating that fit works more as a relational and cultural enabler than as a direct functional value cue.

The mediation results further clarify these mechanisms. The functional value path was only partially supported because expression–product fit did not affect purchase intention through perceived value. By contrast, the affinity path and the expression–culture path were fully supported. Brand warmth transmitted the effects of playfulness, visual attractiveness, and expression–product fit on purchase and sharing intentions. Cultural resonance also transmitted the effects of playfulness, perceived novelty, and expression–product fit on both outcomes. These results indicate that meme-based packaging works especially well when meme expression is able to humanize the brand and create cultural recognition, not merely when it adds surface-level novelty.

### 5.2. Theoretical Implications

This study offers three theoretical implications.

First, the findings clarify how internet memes become effective after they enter packaging design. Meme-based packaging does not influence consumers simply because it contains humorous, trendy, or culturally familiar content. Its effects depend on whether meme expression is translated into packaging cues that consumers can perceive, evaluate, and connect with. In this study, the five stimulus variables represent the consumer-facing outcomes of this translation process. Visual attractiveness strongly increased perceived value, showing that meme expression needs to be organized as a credible visual design rather than placed on the package as a loose cultural reference. Playfulness strengthened brand warmth and cultural resonance, suggesting that humor works when it changes how consumers feel about the brand and how they recognize the cultural meaning of the package. Expression–product fit did not significantly increase perceived value, but it did strengthen brand warmth and cultural resonance. This pattern indicates that fit is less about making the product seem functionally more valuable and more about making the meme expression feel appropriate, culturally intelligible, and naturally integrated into the product context. In this sense, the study theorizes meme-based packaging as a design translation process rather than as a direct transfer of online meme content into a physical medium. This extends recent work on meme marketing and branded memes, which has mainly examined memes as online communication tools or social media brand content, by showing that meme expression needs to be reorganized into consumer-facing packaging cues when it enters a physical product interface (Tsai & Hsiao, 2025; Kim & Baek, 2025; Rathi & Jain, 2024).

Second, the study refines the S-O-R framework by distinguishing between purchase-oriented and sharing-oriented consumer responses. The results show that purchase intention and sharing intention are not driven by the same dominant psychological mechanism. Perceived value was the strongest predictor of purchase intention, whereas cultural resonance was the strongest predictor of sharing intention. Brand warmth supported both outcomes, but it did not replace the distinct roles of value evaluation and cultural recognition. This finding suggests that S-O-R models should not treat behavioral intention as a single, uniform response when the target behaviors have different meanings. In meme-based packaging, purchase intention is closer to product evaluation and commercial conversion, while sharing intention is closer to social communication and cultural expression. By separating these two outcomes, the study provides a more precise explanation of how the same packaging stimulus can support both market evaluation and social circulation through different organism states.

Third, the study contributes to design-oriented consumer research by linking mechanism testing with design feedback. Using IPMA, the results indicate that purchase-oriented design should focus on perceived value and visual attractiveness, whereas sharing-oriented design should focus on cultural resonance, playfulness, and brand warmth. The point is not to add more meme elements, but to match translation priorities to design goals: value and visual credibility for purchase conversion, and cultural recognition and conversational value for social transmission.

### 5.3. Practical Implications

The findings provide several practical implications for packaging designers and brand managers.

First, meme-based packaging should be treated as a translation task rather than a decoration task. The practical question is not whether a package uses a popular meme, but whether the meme expression has been translated into a clear and coherent packaging cue. Designers need to consider whether consumers can recognize the cultural reference, understand the product information, and see the connection between the meme expression and the product. A meme that is simply placed on the package may attract attention, but it may not improve value perception, brand warmth, or cultural resonance.

Second, purchase-oriented meme packaging should protect product value and visual credibility. The results showed that perceived value was the strongest predictor of purchase intention, and IPMA also identified perceived value as the highest-priority construct for purchase intention. This means that playful expression should not weaken the basic product message. Consumers still need to know what the product is, what it offers, and why it is worth buying. Visual attractiveness is also important because it strongly shaped perceived value. For this reason, meme elements should be organized through careful use of layout, typography, color, and imagery, rather than appearing as casual add-ons. This implication is consistent with recent packaging research showing that visual organization, information presentation, packaging value, and creative package design can shape consumer evaluation and purchase-related responses (Dopico-Parada et al., 2021; Liu et al., 2025; Shukla et al., 2022).

Third, sharing-oriented meme packaging should prioritize cultural resonance. Cultural resonance was the strongest predictor of sharing intention and the highest-priority construct in the IPMA for sharing intention. If brands want consumers to photograph, post, discuss, or forward a package, the design should offer more than visual novelty. It should provide a cultural cue that consumers can recognize and feel comfortable showing to others. This may involve humor, familiar platform language, character-based expression, or a culturally recognizable phrase, but these elements need to be used in a way that feels meaningful rather than forced. This also aligns with research on valuable virality, which suggests that people are more likely to share content when it carries emotional relevance, social value, identity expression, or conversational usefulness (Akpinar & Berger, 2017).

Fourth, expression–product fit should be treated as a key design condition. Although expression–product fit did not significantly affect perceived value, it significantly strengthened brand warmth and cultural resonance. This result suggests that fit is not a minor detail. A meme that does not fit the product may still be noticed, but it is less likely to make the brand feel approachable or culturally fluent. Designers should therefore evaluate meme use against the product category, consumption occasion, and brand voice. In practice, the goal is not to add more meme elements, but to make the right meme expression feel naturally connected to the product. For purchase conversion, meme elements should first support value perception and visual credibility; for social circulation, they should first activate cultural resonance and conversational appeal.

### 5.4. Limitations and Future Research

This study has several limitations that provide directions for future research. First, the study used one product context and two packaging stimuli. Although this design helped control the product category, brand information, package format, and basic product information, meme-based packaging may work differently across product categories. Because the stimuli were developed around one focal product context, the findings should be interpreted as evidence of the proposed psychological mechanism rather than as a brand-specific evaluation of Joyoung or the Hakimi product. Future research could examine snacks, beverages, cosmetics, cultural products, or technology products to test whether the proposed Design Translation–S-O-R model remains stable across different consumption contexts. Future research could also examine whether gender, income level, socioeconomic status, and product involvement moderate consumers’ responses to meme-based packaging across different product categories.

Second, the study relied on a cross-sectional survey experiment and self-reported behavioral intentions. The manipulation check confirmed a significant difference between the two stimuli, but the conventional control condition still showed a meme perception mean slightly above the scale midpoint. This likely reflects the difficulty of fully removing culturally playful associations when using a real marketplace brand and product context. Future studies could include a fully neutral non-branded control stimulus, as well as behavioral choice tasks, shelf simulations, eye-tracking, actual purchase records, or real social sharing data to provide stronger behavioral evidence.

Third, the data were collected from Chinese adult consumers through an online questionnaire platform. This sample was appropriate for examining meme-based packaging beyond a single youth segment, but it does not allow strong conclusions about any specific generational cohort because some age groups were relatively small. Meme meanings may also vary across cultures, languages, platforms, and online communities. Future research could use age-balanced or generation-specific samples and compare different cultural contexts to examine how age, digital media experience, and meme familiarity influence consumer responses.

Fourth, meme culture is temporally sensitive and culturally specific. A meme expression that feels fresh and culturally resonant at one moment may quickly become outdated, and the same expression may not generate the same meaning across platforms, regions, or cultural contexts. Future research could compare short-cycle trending memes, long-lasting meme expressions, character-based memes, and event-based memes, as well as cross-cultural samples, to examine how meme temporality and cultural specificity affect packaging evaluation and sharing intention.

Fifth, common method bias remains a methodological consideration because the data were collected through a single self-report questionnaire. Although the CFA model comparison and full collinearity diagnostics suggested that a single common method factor was unlikely to fully explain the observed relationships, future studies could reduce this concern further through temporal separation, multi-source data, longitudinal designs, or behavioral outcome measures.

## 6. Conclusions

This study examined how meme-based packaging influences purchase intention and sharing intention through perceived value, brand warmth, and cultural resonance. The findings show that meme-based packaging does not work through entertainment alone. Purchase intention was mainly driven by perceived value, whereas sharing intention was mainly driven by cultural resonance. Brand warmth also supported both outcomes, suggesting that playful and well-integrated meme expression can make a brand feel more human, approachable, and socially engaging.

The study advances a design translation view of meme-based packaging. Internet memes do not become effective packaging cues simply by being placed on a product surface. They need to be translated into design elements that remain clear, visually attractive, novel, and relevant to the product context. When this translation is successful, packaging may function not only as a commercial interface for product evaluation, but also as a social medium that carries recognizable digital cultural meaning.

For scholars, this study connects meme marketing, packaging design, and the S-O-R framework by showing how digital cultural symbols can be transformed into consumer-facing packaging cues and how these cues activate different psychological mechanisms. In doing so, the study responds to recent calls for closer attention to packaging as a multidimensional driver of consumer perception and behavior, while also extending meme-marketing research beyond online advertising and social media communication. For practitioners, the findings suggest that effective meme-based packaging should balance value communication, visual quality, brand warmth, and cultural resonance rather than simply adding more internet jokes or trendy symbols.

## Figures and Tables

**Table 1:** Theoretical foundations and cue-route mapping in the proposed model.

Route or Perspective	Constructs or Paths Supported	Role in This Study	Key References
Functional value route	Information clarity, visual attractiveness, expression–product fit → perceived value	Packaging design, perceived value, and schema congruity explain how clear information, visual quality, and fit support product evaluation.	Zeithaml (1988); Orth and Malkewitz (2008); Bloch (1995); Meyers-Levy and Tybout (1989)
Affinity–image route	Playfulness, visual attractiveness, expression–product fit → brand warmth	Hedonic consumption and brand relationship theory explain how playful and well-integrated package expression can make the brand feel friendly and approachable.	Holbrook and Hirschman (1982); Aaker et al. (2010); Kervyn et al. (2012); Fournier (1998)
Expression–culture route	Playfulness, perceived novelty, expression–product fit → cultural resonance	Meme marketing, symbolic consumption, and social transmission theory explain why humorous, novel, and fitting digital cultural cues can become socially meaningful.	Shifman (2014); Tsai and Hsiao (2025); Kim and Baek (2025); Berger and Milkman (2012)
Outcome differentiation	Perceived value, brand warmth, cultural resonance → purchase intention and sharing intention	The S-O-R framework integrates design stimuli, organism states, and behavioral responses while allowing purchase and sharing to follow different psychological logics.	Mehrabian and Russell (1974); Ajzen (1991); Berger (2014)

**Table 2:** Data screening procedure.

Screening Step	Number
Initial responses collected	358
Excluded responses	53
Final valid responses	305
Valid response rate	85.20%
Meme-based packaging condition	151
Conventional packaging control condition	154

**Table 3:** Sample profile and group distribution (N = 305).

Variable	Category	n	%
Group	Meme-based packaging	151	49.5
Group	Conventional control	154	50.5
Gender	Female	183	60.0
Gender	Male	80	26.2
Gender	Not disclosed	42	13.8
Age	18–24	8	2.6
Age	25–30	89	29.2
Age	31–40	113	37.0
Age	41–50	86	28.2
Age	51+	9	3.0
Education	High school or below	44	14.4
Education	Junior college	118	38.7
Education	Bachelor’s degree	119	39.0
Education	Master’s degree or above	24	7.9
Purchase frequency	1–2 times/week	79	25.9
Purchase frequency	3–4 times/week	97	31.8
Purchase frequency	5+ times/week	129	42.3

**Table 4:** Measurement constructs and main sources.

Construct	Abbreviation	No. of Items	Main Source Domains
Packaging playfulness	PP	3	Experiential consumption and perceived playfulness (Holbrook & Hirschman, 1982; Moon & Kim, 2001)
Information clarity	IC	3	Packaging communication and information quality (Silayoi & Speece, 2007; DeLone & McLean, 2003)
Visual attractiveness	VA	3	Product design and visual attractiveness (Bloch, 1995; Lavie & Tractinsky, 2004)
Perceived novelty	PN	3	Creative packaging and product novelty (Shukla et al., 2022; Magnier et al., 2016)
Expression–product fit	EPF	3	Schema congruity and perceived fit (Meyers-Levy & Tybout, 1989; Becker-Olsen et al., 2006)
Perceived value	PV	3	Perceived value and value measurement (Zeithaml, 1988; Sweeney & Soutar, 2001)
Brand warmth	BW	3	Brand warmth and intentional-agent perception (Aaker et al., 2010; Kervyn et al., 2012)
Cultural resonance	CR	3	Symbolic consumption and cultural-product response (Belk, 1988; Li et al., 2023)
Purchase intention	PI	3	Product evaluation and purchase-intention measurement (Dodds et al., 1991; Spears & Singh, 2004)
Sharing intention	SI	3	Word-of-mouth and online sharing behavior (Berger & Milkman, 2012; Chu & Kim, 2011)

**Table 5:** Manipulation check.

Condition	n	M	SD	Test Statistic	*p*-Value	Effect Size
Meme	151	5.31	1.14	—	—	—
Control	154	4.14	1.22	t(303) = 8.64	<0.001	d = 0.99

**Table 6:** Reliability and convergent validity.

Construct	Cronbach’s Alpha	rho_a	rho_c	AVE
BW	0.909	0.909	0.943	0.846
CR	0.913	0.913	0.945	0.851
EPF	0.906	0.907	0.941	0.842
IC	0.913	0.916	0.945	0.852
PI	0.925	0.925	0.952	0.869
PN	0.919	0.920	0.949	0.861
PP	0.901	0.902	0.938	0.835
PV	0.922	0.923	0.951	0.866
SI	0.925	0.926	0.952	0.870
VA	0.908	0.909	0.942	0.844

**Table 7:** Structural model results.

Hypothesis	Path	Beta	t	*p*	f^2^	Result
H1a	IC → PV	0.226	3.953	<0.001	0.062	Supported
H1b	VA → PV	0.572	13.266	<0.001	0.474	Supported
H1c	EPF → PV	0.068	1.323	0.186	0.005	Not supported
H2	PV → PI	0.413	7.281	<0.001	0.186	Supported
H4a	PP → BW	0.431	8.179	<0.001	0.236	Supported
H4b	VA → BW	0.308	5.154	<0.001	0.111	Supported
H4c	EPF → BW	0.147	3.403	0.001	0.033	Supported
H5a	BW → PI	0.185	3.320	0.001	0.037	Supported
H5b	BW → SI	0.289	4.513	<0.001	0.087	Supported
H7a	PP → CR	0.405	7.188	<0.001	0.201	Supported
H7b	PN → CR	0.349	6.460	<0.001	0.150	Supported
H7c	EPF → CR	0.146	3.357	0.001	0.036	Supported
H8a	CR → PI	0.289	5.176	<0.001	0.092	Supported
H8b	CR → SI	0.511	9.028	<0.001	0.272	Supported

**Table 8:** Mediation hypotheses and specific indirect effects.

Hypothesis	Indirect Path	Beta	t	*p*	Result
H3a	IC → PV → PI	0.093	3.529	<0.001	Supported
H3b	VA → PV → PI	0.237	6.084	<0.001	Supported
H3c	EPF → PV → PI	0.028	1.296	0.195	Not supported
H6a	PP → BW → PI	0.080	3.165	0.002	Supported
H6b	PP → BW → SI	0.124	4.135	<0.001	Supported
H6c	VA → BW → PI	0.057	2.503	0.012	Supported
H6d	VA → BW → SI	0.089	3.043	0.002	Supported
H6e	EPF → BW → PI	0.027	2.304	0.021	Supported
H6f	EPF → BW → SI	0.043	2.544	0.011	Supported
H9a	PP → CR → PI	0.117	4.587	<0.001	Supported
H9b	PP → CR → SI	0.207	5.483	<0.001	Supported
H9c	PN → CR → PI	0.101	3.671	<0.001	Supported
H9d	PN → CR → SI	0.178	5.024	<0.001	Supported
H9e	EPF → CR → PI	0.042	2.567	0.010	Supported
H9f	EPF → CR → SI	0.074	3.175	0.002	Supported

**Table 9:** IPMA priorities.

Target Outcome	Construct	Importance	Performance	Priority
PI	PV	0.413	61.976	Highest priority
PI	VA	0.294	61.411	High priority
PI	CR	0.289	61.939	High priority
PI	PP	0.197	62.737	Medium priority
PI	BW	0.185	61.544	Medium priority
SI	CR	0.511	61.939	Highest priority
SI	PP	0.331	62.737	High priority
SI	BW	0.289	61.544	High priority
SI	PN	0.178	62.784	Medium priority

## Data Availability

The anonymized dataset, questionnaire materials, and schematic stimulus illustrations supporting the findings of this study are available from the corresponding author upon reasonable request. The original commercial package images are not redistributed because of copyright restrictions.

## Supplementary Materials

The following supporting information can be downloaded at https://www.mdpi.com/article/10.3390/bs16060972/s1, Table S1, measurement items; Table S2, control variable coding; Table S3, HTMT matrix; Table S4, common method bias diagnostics; Table S5, construct score robustness check.
