# Supplementary material for: Meme-Based Packaging as Digital Cultural Translation: How Online Cultural Symbols Shape Purchase and Sharing Intentions
Source: Behav Sci (Basel). 2026 Jun 11;16(6):972. doi: 10.3390/bs16060972 (PMC13296252; doi:10.3390/bs16060972)
Supplement: Supplementary file 1 [file behavsci-16-00972-s001.zip › behavsci-4324756-supplementary.pdf]

Supplementary Materials

Meme-Based Packaging as Digital Cultural Translation: How Online Cultural Symbols Shape Purchase and Sharing Intentions

Table S1. Measurement items and item sources.

Construct	Code	Item wording	Main sources
Packaging playfulness	PP1	This package looks humorous and entertaining.	
Packaging playfulness	PP2	This package gives me a lighthearted and playful feeling.	Holbrook and Hirschman (1982); Moon and Kim (2001)
Packaging playfulness	PP3	The expression style of this package makes the product feel more fun.	
Information clarity	IC1	The product information on this package is easy to understand.	
Information clarity	IC2	The package clearly communicates what the product is.	Silayoi and Speece (2007); DeLone and McLean (2003)
Information clarity	IC3	The playful expression does not prevent me from understanding the product information.	
Visual attractiveness	VA1	The visual design of this package is attractive.	
Visual attractiveness	VA2	The graphics, color, and layout of this package look visually pleasing.	Bloch (1995); Lavie and Tractinsky (2004)
Visual attractiveness	VA3	Overall, this package has a good visual appearance.	
Perceived novelty	PN1	This package looks different from ordinary product packaging.	
Perceived novelty	PN2	The expression style of this package feels fresh and novel.	Shukla et al. (2022); Magnier et al. (2016)
Perceived novelty	PN3	This package gives me a sense of originality.	
Expression–product fit	EPF1	The expression style of this package fits the product well.	
Expression–product fit	EPF2	The meme-like elements are appropriate for this product category.	Meyers-Levy and Tybout (1989); Becker-Olsen et al. (2006)
Expression–product fit	EPF3	The playful expression is consistent with the product and its consumption context.	
Perceived value	PV1	This product seems worth considering.	
Perceived value	PV2	This product appears to offer good value.	Zeithaml (1988); Sweeney and Soutar (2001)
Perceived value	PV3	Overall, this package increases my perception of the product’s value.	
Brand warmth	BW1	This package makes the brand seem friendly.	
Brand warmth	BW2	This package makes the brand feel approachable.	Aaker et al. (2010); Kervyn et al. (2012)
Brand warmth	BW3	This package makes the brand seem more sincere and human.	

Construct	Code	Item wording	Main sources
Cultural resonance	CR1	I can recognize the digital cultural expression conveyed by this package.	
Cultural resonance	CR2	This package connects with a cultural or internet context that feels familiar to me.	McCracken (1986); Belk (1988); Holt (1995); Li et al. (2023)
Cultural resonance	CR3	The expression of this package gives me a sense of shared cultural meaning.	
Purchase intention	PI1	I would consider buying this product.	
Purchase intention	PI2	I would be willing to try this product.	Dodds et al. (1991); Spears and Singh (2004)
Purchase intention	PI3	If I saw this product in a store or online, I might choose it.	
Sharing intention	SI1	I would be willing to share this package with others.	
Sharing intention	SI2	I would be willing to take a photo of this package or discuss it with others.	Berger and Milkman (2012); Chu and Kim (2011)
Sharing intention	SI3	I would be willing to recommend or forward information about this package online or offline.	
Manipulation check	MC1	This package contains obvious internet-meme or online-culture elements.	Developed for this study based on stimulus-manipulation logic
Manipulation check	MC2	The expression style of this package feels strongly meme-based.	
Meme familiarity	MF1	I am familiar with internet memes and online slang.	
Meme familiarity	MF2	I often understand popular meme-based expressions on social media.	Shifman (2014); Tsai and Hsiao (2025); Kim and Baek (2025)
Meme familiarity	MF3	I usually pay attention to internet memes and digital popular-culture expressions.	

Note. All items were measured on seven-point Likert scales, ranging from 1 = strongly disagree to 7 = strongly agree. PP = packaging playfulness; IC = information clarity; VA = visual attractiveness; PN = perceived novelty; EPF = expression–product fit; PV = perceived value; BW = brand warmth; CR = cultural resonance; PI = purchase intention; SI = sharing intention; MC = manipulation check; MF = meme familiarity. Meme familiarity was used only in the supplementary moderation analysis and was therefore reported as a supplementary construct rather than as part of the focal ten-construct measurement model.

Table S2. Control-variable coding.

Variable	Coding type	Description	Use in analysis
Age	Ordinal category	1 = 18–24; 2 = 25–30; 3 = 31–40; 4 = 41–50; 5 = 51 or above	Included in the supplementary robustness check
Education	Ordinal category	1 = high school or below; 2 = junior college; 3 = bachelor’s degree; 4 = master’s degree or above	Included in the supplementary robustness check
Purchase frequency	Ordinal category	1 = 1–2 times/week; 2 = 3–4 times/week; 3 = 5 or more times/week	Included in the supplementary robustness check
Price sensitivity	Likert-type item	Item wording: When purchasing beverages, snacks, or similar fast-moving consumer goods, how sensitive are you to price? Scale: 1 = not sensitive at all, 5 = very sensitive.	Included in the supplementary robustness check
Brand familiarity	Likert-type item	Item wording: Before participating in this survey, how familiar were you with this brand? Scale: 1 = very unfamiliar, 7 = very familiar.	Included in the supplementary robustness check
Prior purchase experience	Binary item	Item wording: Have you previously purchased related products from this brand? Scale: never / rarely / occasionally / many times; recoded for robustness checks.	Included in the supplementary robustness check
Gender	Optional demographic item	Female; male; not disclosed	Reported descriptively and included in the supplementary robustness check as a background control

Note. The robustness check included seven background variables: age, gender, education, purchase frequency, price sensitivity, brand familiarity, and prior purchase experience. These variables were used only for supplementary robustness analysis and were not treated as core theoretical variables.

Table S3. HTMT matrix.

Construct	PP	IC	VA	PN	EPF	PV	BW	CR	PI	SI
PP	—									
IC	0.610	—								
VA	0.761	0.628	—							
PN	0.787	0.639	0.774	—						
EPF	0.628	0.759	0.680	0.620	—					
PV	0.809	0.653	0.811	0.809	0.630	—				
BW	0.803	0.646	0.765	0.769	0.640	0.809	—			
CR	0.813	0.626	0.768	0.787	0.630	0.799	0.810	—		
PI	0.802	0.642	0.783	0.803	0.665	0.826	0.768	0.793	—	
SI	0.745	0.625	0.784	0.770	0.630	0.787	0.726	0.788	0.793	—

Note. All HTMT values were below the conservative threshold of 0.85. The highest value was observed between purchase intention and perceived value, HTMT = 0.826.

Table S4. Common method bias diagnostics.

Test	Result	Interpretation
Harman's single-factor test	The first unrotated factor explained 60.25% of the total variance.	The result suggested a potential common method concern and required additional diagnostics.
Full collinearity VIF	The values were 3.145 for PP, 2.257 for IC, 3.078 for VA, 3.191 for PN, 2.314 for EPF, 3.733 for PV, 3.107 for BW, 3.333 for CR, 3.512 for PI, and 3.039 for SI. All values were below 5, although several approached or exceeded the stricter 3.3 criterion. Therefore, common method bias was interpreted cautiously rather than dismissed.	All values were below 5; several approached or exceeded the stricter 3.3 criterion, so common method bias was interpreted cautiously.
One-factor CFA model	χ^2 = 2332.519, df = 405, χ^2/df = 5.759, CFI = 0.786, TLI = 0.771, RMSEA = 0.125, SRMR = 0.062	Poor model fit.
Ten-factor CFA model	χ^2 = 401.124, df = 360, χ^2/df = 1.114, CFI = 0.995, TLI = 0.994, RMSEA = 0.019, SRMR = 0.022	Substantially better fit than the one-factor model.

Note. Harman's single-factor test was used as an initial diagnostic only. Because the first factor explained 60.25% of the total variance, full collinearity VIF and CFA model comparison diagnostics were also reported. The full collinearity VIF values were all below 5, although several approached or exceeded the stricter 3.3 criterion. The ten-factor measurement model fitted the data substantially better than the one-factor model, indicating that a single common method factor did not adequately explain the covariance structure among the focal measurement items.

Table S5. Construct-score robustness check with control variables.

Outcome variable	Model specification	R^2	Key focal relationship	Pattern after adding controls	Interpretation
Purchase intention	Focal predictors only	0.657	Perceived value → purchase intention	Positive and significant	Perceived value was the strongest predictor of purchase intention in the focal model.
Purchase intention	Focal predictors + controls	0.666	Perceived value → purchase intention	Remained positive and significant	The effect remained stable after adding age, gender, education, purchase frequency, price sensitivity, brand familiarity, and prior purchase experience.
Purchase intention	Focal predictors + controls	0.666	Brand warmth → purchase intention	Remained positive and significant	The relationship was stable but weaker than the perceived-value route.
Purchase intention	Focal predictors + controls	0.666	Cultural resonance → purchase intention	Remained positive and significant	Cultural resonance continued to contribute to purchase intention after controls were added.
Sharing intention	Focal predictors only	0.563	Cultural resonance → sharing intention	Positive and significant	Cultural resonance was the strongest predictor of sharing intention in the focal model.
Sharing intention	Focal predictors + controls	0.572	Cultural resonance → sharing intention	Remained positive and significant	The effect remained stable after adding the seven background variables.
Sharing intention	Focal predictors + controls	0.572	Brand warmth → sharing intention	Remained positive and significant	Brand warmth continued to support sharing intention, although cultural resonance remained the dominant mechanism.
Purchase intention	Focal predictors + controls	0.666	Expression–product fit → perceived value	Remained not significant	The non-significance of this path remained consistent, supporting the interpretation that expression–product fit worked more through brand warmth and cultural resonance than through perceived value.

Note. The robustness check added seven background variables: age, gender, education, purchase frequency, price sensitivity, brand familiarity, and prior purchase experience. The R^2 value for purchase intention increased from 0.657 to 0.666, and the R^2 value for sharing intention increased from 0.563 to 0.572. The focal relationships remained substantively unchanged.
